# Upregulation of rate-limiting enzymes in cholesterol metabolism by PKCδ mediates endothelial apoptosis in diabetic wound healing

**DOI:** 10.1038/s41420-024-02030-2

**Published:** 2024-05-29

**Authors:** Peiliang Qin, Peng Zhou, Yating Huang, Binbin Long, Ruikang Gao, Shan Zhang, Bingjie Zhu, Yi-Qing Li, Qin Li

**Affiliations:** 1 Department of Vascular Surgery, Union Hospital, Tongji Medical College, Huazhong University of Science and Technology, Wuhan, Hubei China; 2 General Surgery Department, Taihe Hospital Affiliated to Hubei University of Medicine, Shiyan, Hubei China

**Keywords:** Cell signalling, Cell death

## Abstract

Diabetic foot ulcer (DFU) is a prevalent complication of diabetes that poses significant challenges in terms of treatment and management. It is characterized by heightened endothelial apoptosis and impaired angiogenesis. In this study, we aimed to investigate the role of protein kinase Cδ (PKCδ) in regulating endothelial apoptosis in diabetic wounds by promoting cholesterol biosynthesis. The expression of PKCδ was increased in human umbilical vascular endothelial cells (HUVECs) cultivated in high glucose medium and skin tissue isolated from diabetic mice. High glucose-induced HUVECs apoptosis was reduced by PKCδ inhibition with siRNA or rottlerin. RNA-seq identified two enzymes, 3-hydroxy-3-methylglutaryl-CoA synthase 1 (HMGCS1) and 3-hydroxy-3-methylglutaryl-CoA reductase (HMGCR), as the downstream of PKCδ. PKCδ knockdown or inhibition suppressed the expression of HMGCS1 and HMGCR and lowered free cholesterol (FC) levels. Cholesterol restored high glucose-induced apoptosis in siRNA- or rottlerin-treated HUVECs. In vivo use of rosuvastatin calcium, an inhibitor of HMGCR, downregulated free cholesterol levels and accelerated the wound healing process. In conclusion, PKCδ expression in endothelial cells was activated by high glucose, which subsequently upregulates the expression of two enzymes catalyzing cholesterol biosynthesis, HMGCS1 and HMGCR. Enhanced cholesterol biosynthesis raises free cholesterol levels, promotes endothelial apoptosis, and finally delays wound healing.

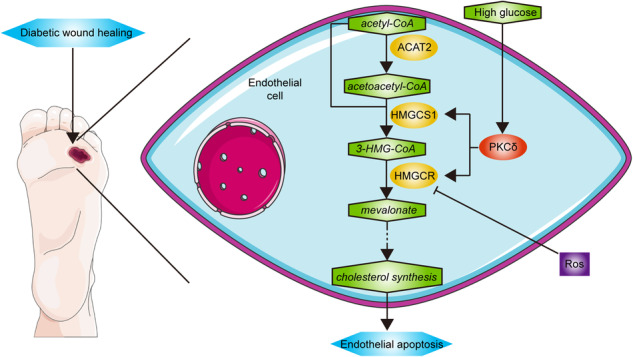

## Introduction

Diabetes mellitus (DM), characterized by elevated blood glucose, has become a bigger challenge over the years. Patients with diabetes are particularly affected by long-term complications, including macrovascular complications and microvascular complications [1]. One manifestation of microvascular complications is diabetic foot ulcers (DFUs), which disturbs 15-25% of individuals with diabetes [2]. DFUs typically present as painful ulcers that occur in areas of skin affected by neuropathy and calluses, such as the plantae and tips of the toes. The pathogenesis of DFUs involves unrecognized trauma, significantly delayed healing, and inflammation. Severe comorbidities, including infections and osteomyelitis, occur in 25% of patients with DFU, often leading to amputation and a high mortality rate.

Hemostasis, inflammation, proliferation, and remodeling are four interconnected stages of wound healing [3, 4]. Immediately after an injury, the coagulation system is activated to control blood loss and prevent microbial invasion. Subsequently, pro-inflammatory cells, such as neutrophils and macrophages, accumulate at the wound site and wound debridement was performed. Following this, keratinocytes, fibroblasts, and endothelial cells migrate and proliferate, promoting epidermal resurfacing, fibroplasia, angiogenesis, extracellular matrix (ECM) deposition, and wound contraction. In the final stage of remodeling, the tensile strength of ECM increases and blood supply to the area decreases. However, in diabetes, the balance between growth, proliferation, maturation, and quiescence of blood vessels is disrupted, inhibiting angiogenesis and perturbing the normal wound healing process [5]. In contrast to diabetic retinopathy and diabetic nephropathy, DFU is characterized by reduced angiogenesis. On one hand, high glucose levels damage the integrity of endothelial cells, promoting their apoptosis, detachment, and circulation into the bloodstream. On the other hand, DM also decreases pro-angiogenic factors while increasing anti-angiogenic factors.

Protein kinase C (PKC) is a group of serine/threonine-related protein kinases belonging to the AGC (cAMP-dependent protein kinase/PKG/PKC) family. Several PKC isoforms including PKCα, PKCβ, PKCδ, PKCε, and PKCζ have been detected to be activated in vascular tissues and cell types under diabetic state [6]. Protein Kinase Cδ (PKCδ) is an isoform of the PKC family that belongs to the novel PKC (nPKC) group, which is Ca^2+^-independent and activated by phospholipids and DAG. It increases insulin resistance and mediates the progression of diabetic complications including atherosclerosis, diabetic cardiomyopathy, diabetic foot ulcer, diabetic retinopathy, and diabetic nephropathy. However, the mechanism of PKCδ in regulating diabetic wound healing has not been fully elucidated [7–12]. Increased expression of PKCδ has been observed in diabetic fibroblasts, and inhibiting PKCδ has been shown to improve wound healing. Additionally, diabetic mice exhibited decreased expression of pro-angiogenic factors such as vascular endothelial growth factor (VEGF) and platelet-derived growth factor (PDGF), along with poor collateral vessel formation, whereas these changes were normalized in diabetic PKCδ-knockout mice [13]. Hyperglycemia-induced PKCδ activation also enhanced vascular cell apoptosis and acellular capillaries formation in diabetic mouse retinas [11]. Therefore, it is reasonable to propose that high glucose-induced PKCδ expression leads to the apoptosis of endothelial cells, impairing angiogenesis and delaying wound healing.

However, the underlying mechanism is still under investigation. Although endothelial cells are relatively inactive in lipid metabolism, some studies have revealed that they maintain cholesterol hemostasis by regulating cholesterol biosynthesis and lipoprotein transportation [14, 15]. The accumulation of free cholesterol has been demonstrated to cause cell apoptosis [16–18]. Deletion of PKCδ has been found to improve insulin sensitivity in mice fed a high-fat diet and reduce lipid loading in mice with nonalcoholic steatohepatitis [19, 20]. In this study, we have uncovered a novel pathway by which PKCδ mediates high glucose-induced endothelial apoptosis. High glucose upregulates PKCδ, which subsequently increases the expression of HMGCS1 and HMGCR, leading to cholesterol synthesis, endothelial cell apoptosis, and delayed wound healing.

## Results

### PKCδ was elevated in high glucose-treated HUVECs and the skin of type 2 diabetes mice

**Fig. 1:**
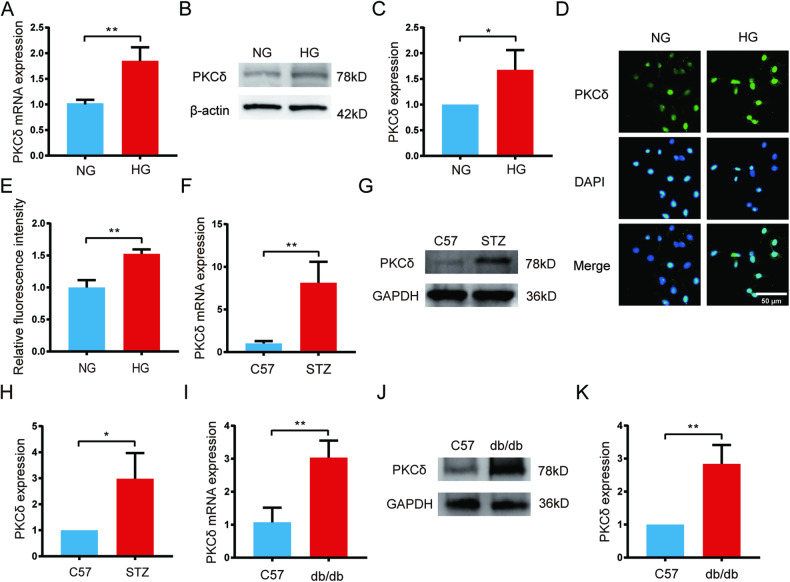
PKCδ was elevated in high glucose-treated HUVECs and the skin of type 2 diabetes mice. **A** mRNA level of PKCδ was determined using qPCR after treatment with high glucose (22.5 mM) for 48 h respectively. **B**, **C** Protein expression detection with western blot and quantification of PKCδ after HG treatment for 72 h. **D**, **E** PKCδ detection by immunofluorescence and quantification of fluorescence intensity after HG treatment for 24 h. **F** mRNA level of PKCδ in skin tissues of STZ-induced diabetes mice. **G**, **H** Protein level and quantification of PKCδ in skin tissues of STZ-induced diabetes mice. **I** mRNA expression of PKCδ in skin tissues isolated from db/db mice. **J**, **K** Protein expression and quantification of PKCδ in skin tissues isolated from db/db mice. Each experiment was replicated at least thrice, and representative images were shown. Quantification was done using a two-tailed unpaired Student’s *t* test. The results are presented as the mean ± SEM, *p < 0.05, **p < 0.01 vs controls.

Previous studies have demonstrated the activation of PKCδ in various cell types under high glucose conditions [21–23]. To clarify the effect of high glucose (HG) on PKCδ expression in HUVECs, we exposed the cells to DMEM medium with normal glucose (5.5 mM) or high glucose (22.5 mM). Total RNA was extracted following 48 h treatment and qPCR was performed to analyze PKCδ mRNA expression (Fig. 1A). The HG-treated group showed a significant increase in PKCδ mRNA levels, with a 1.8-fold change compared to the normal glucose (NG) group. Furthermore, protein analysis using western blotting showed elevated levels of PKCδ in HUVECs after HG treatment (Fig. 1B, C). Immunofluorescence analysis also revealed higher PKCδ immunofluorescence intensity in the HG group after 72 h of treatment (Fig. 1D, E). confirming the upregulation of PKCδ in high glucose-treated HUVECs.

Diabetic foot ulcers, a complex consequence of diabetes, involve the dysfunction of multiple cell types, including endothelial cells, keratinocytes, and fibroblasts [4]. To investigate the influence of chronically elevated blood glucose on the expression of PKCδ in skin tissues, we used two types of type 2 diabetes mouse models. Two weeks after the diabetes model formation, we extracted skin tissues from STZ-induced diabetes mice. RNA and protein were extracted using tissue grinders. Despite some variation, we found higher levels of PKCδ mRNA and protein in the skin tissues of STZ-induced diabetic mice compared to age- and weight-matched control C57 mice (Fig. 1F–H and Supplementary Fig. S4A). Similarly, skin tissues from db/db mice, harvested after adaptive feeding for two weeks, showed a 3-fold increase in PKCδ mRNA levels and a 2.8-fold increase in PKCδ protein levels compared to normal C57 mice (Fig. 1I–K and Supplementary Fig. S4B). These findings provide evidence of increased PKCδ expression in diabetic skin tissues, suggesting its potential contribution to diabetic foot ulcers.

### PKCδ knockdown by siRNA ameliorated high glucose-induced HUVECs apoptosis

**Fig. 2:**
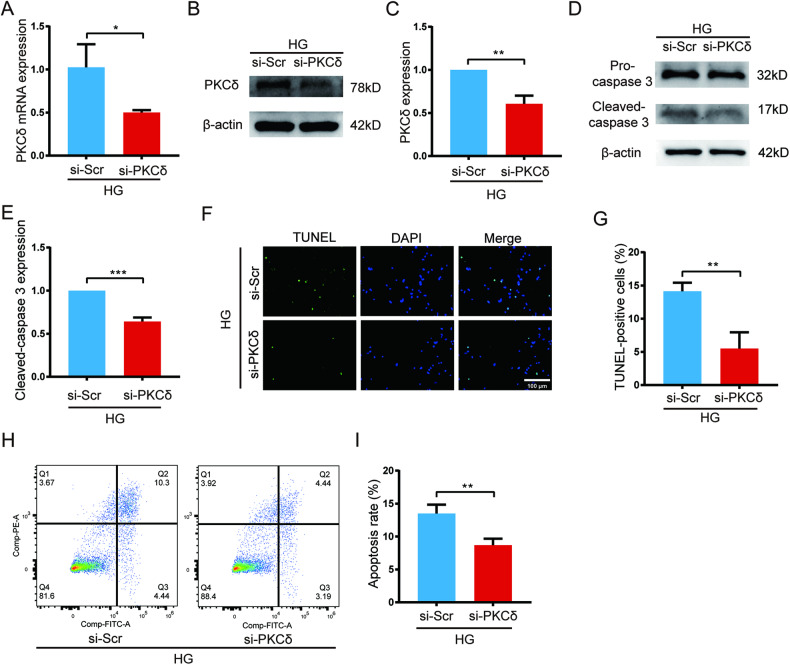
PKCδ knockdown by siRNA ameliorated high glucose-induced HUVECs apoptosis. **A** qPCR analysis of mRNA level of PKCδ after si-PKCδ transfection for 48 h. **B**, **C** Western blot and quantification of PKCδ protein level after transfection for 72 h. **D**, **E** The protein level of pro-caspase 3 and cleaved-caspase 3 and quantification of cleaved-caspase 3 72 h after transfection. **F**, **G** TUNEL assay and quantification of TUNEL-positive cells 72 h after transfection. **H**, **I** Flow cytometry assay and quantification of apoptotic cells 72 h after transfection. Each experiment was replicated for at least thrice and representative images were shown. Quantification was done using a two-tailed unpaired Student’s *t* test. The results are presented as the mean ± SEM, *p < 0.05, **p < 0.01 vs controls.

High glucose has been demonstrated to induce apoptosis of not just endothelial cells but many other types of cells [24–27]. In several studies, PKCδ has been demonstrated to be pro-apoptotic [28–30]. We may propose that PKCδ mediates high glucose-induced HUVECs apoptosis. HUVECs were transfected with si-PKCδ and cultured for 48 h before RNA extraction. qPCR showed a 50% decrease of PKCδ mRNA compared with the si-PKCδ group (Fig. 2A). The protein level was detected 72 h after transfection by western blot, which showed a 40% reduction (Fig. 2B, C). These results validated our siRNA. To clarify whether PKCδ mediates high glucose-induced apoptosis, we transfected si-PKCδ into HUVECs cultured under high glucose. Apoptosis assays were implemented 72 h after transfection. Western blot showed a 36% decrease of caspase 3 cleavage in the PKCδ knockdown group compared to the control group (Fig. 2D, E). In addition, the TUNEL assay exhibited a decrease of the percentage of apoptotic cells from 14 to 5.5% after PKCδ inhibition (Fig. 2F, G). Besides, the si-PKCδ group also displayed fewer apoptotic cells (early apoptotic cells plus late apoptotic cells) by flow cytometry (Fig. 2H, I). These results implied that PKCδ facilitates high glucose-induced HUVECs apoptosis.

### PKCδ inhibition by rottlerin ameliorated high glucose-induced HUVECs apoptosis

Rottlerin (mallotoxin), is a small molecular natural compound isolated from *Mallotus Philippinensis*, has been widely used as an effective PKCδ inhibitor in previous studies [31–33]. To further validate the inhibition of PKCδ on cell apoptosis under high glucose, HUVECs were treated with rottlerin at a concentration of 5 μM for 48 h. The control group received an equal amount of solvent (DMSO). Similar to the effect observed with si-PKCδ, rottlerin treatment reduced the expression of cleaved-caspase 3 by 40% (Supplementary Figs. S1A, B). The percentage of TUNEL-positive cells was also reduced from 14% to 5% (Supplementary Figs. S1C, D). Moreover, the percentage of FITC-positive cells, which represents all apoptotic cells, returned to normal levels (Supplementary Figs. S1E, F). These results indicate that PKCδ inhibition also ameliorates high glucose-induced apoptosis in HUVECs.

### RNA-seq and bioinformatic analysis

**Fig. 3:**
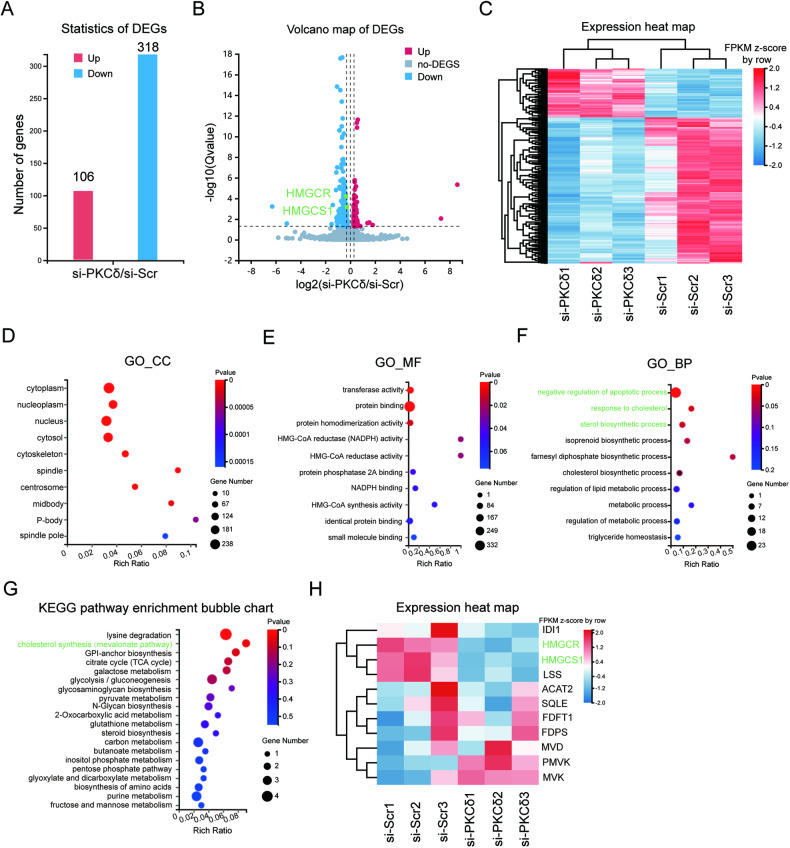
RNA-seq and bioinformatic analysis. **A** The number of DEGs satisfying fold changes ≥1.2 and *q* value ≤ 0.05. **B**, **C** The volcano map and heat map of 424 DEGs. Two genes colored green were chosen for subsequent study. **D**–**F** GO (GO_CC, GO_MF, GO_BP) analysis of 424 DEGs. Significantly enriched biological pathways were colored green. **G** KEGG pathway enrichment of 424 DEGs. Enriched metabolic pathways were colored green. **H** The heat map of genes encoding the enzymes catalyzing the cholesterol synthesis pathway. Two genes colored green were significantly differentially expressed in two groups.

**Fig. 4:**
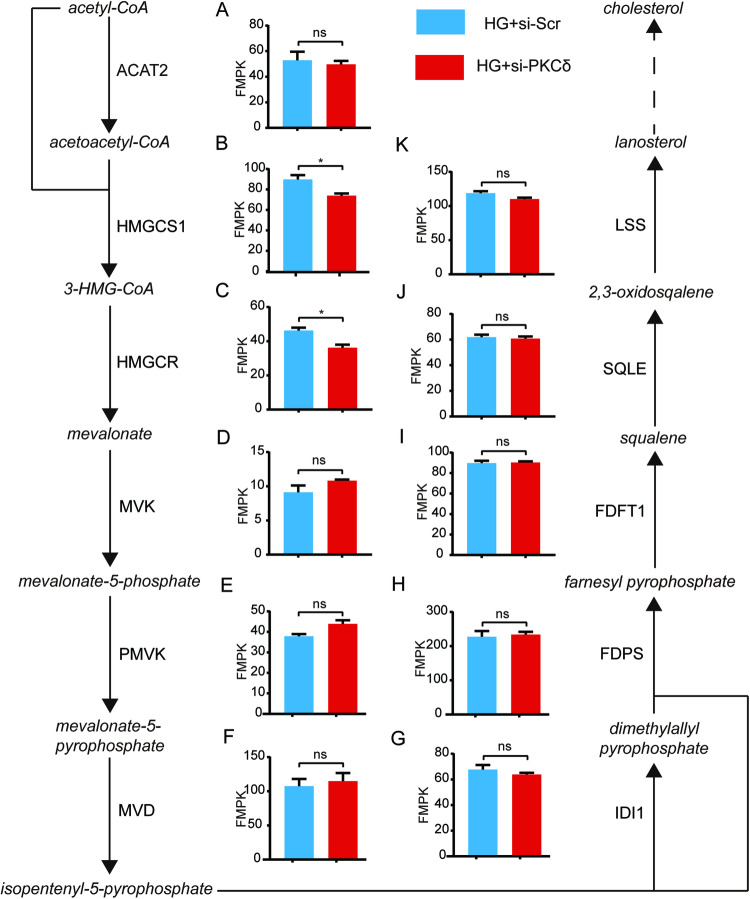
The flowchart of the cholesterol synthesis process and the expression of genes encoding enzymes catalyzing the process. **A** ACAT2; **B** HMGCS1; **C** HMGCR; **D** MVK; **E** PMVK; **F** MVD; **G** IDI1; **H** FDPS; **I** FDFT1; **J** SQLE; **K** LSS. Genes colored green were differentially expressed in two groups and selected for subsequent detection. Quantification was done using the Dr. TOM platform. The results are presented as the mean ± SEM, *q < 0.05 vs controls; ns not significant.

Based on the aforementioned findings, it can be inferred that PKCδ plays a role in mediating the apoptosis of HUVECs induced by high glucose. To further elucidate the downstream molecular mechanism following PKCδ activation, an RNA-seq analysis was conducted. In the experimental group, si-PKCδ was transfected into HUVECs exposed to a high glucose environment, while si-Scr was transfected into the control group. Each group contained three replicates and total RNA was extracted 72 h after transfection. RNA quality was assessed before RNA-seq implementation. Data analysis was done on the Dr. TOM approach, an online BGI database. We finally got 486 differentially expressed genes (DEGs) with the threshold of *q* value ≤ 0.05 or FDR ≤ 0.001. Among these, 424 DEGs were selected based on fold changes ≥1.2, with 106 genes upregulated in the si-PKCδ group and 318 genes downregulated (Fig. 3A). Volcano and heat maps were generated to visualize the expression patterns of the 424 DEGs (Fig. 3B, C). Furthermore, gene ontology (GO) analysis, including cellular component analysis, molecular function analysis, and biological process analysis, was also done (Fig. 3D–F). The DEGs were significantly enriched in the regulation of apoptosis and cholesterol metabolism (*p* < 0.05). Additionally, KEGG enrichment was implemented and metabolic pathways, especially lipid metabolism pathways, were focused on (Fig. 3G). Notably, the mevalonate pathway, which regulates cholesterol synthesis, was found to be the most significantly enriched lipid metabolism pathway. In the si-PKCδ group, two important enzymes involved in this pathway, namely 3-hydroxy-3-methylglutaryl-CoA reductase (HMGCR) and 3-Hydroxy-3-Methylglutaryl-CoA Synthase 1 (HMGCS1), were downregulated. HMGCR catalyzes the rate-limiting step from 3-hydroxy-3-methylglutaryl-CoA (3-HMG-CoA) to mevalonate during cholesterol biosynthesis, while HMGCS1 catalyzes the preceding step from acetoacetyl-CoA to 3-HMG-CoA. A heat map was generated to visualize the transcriptional expressions of the genes encoding enzymes catalyzing cholesterol synthesis (Fig. 3H). Additionally, a flowchart was created to illustrate the process of cholesterol biosynthesis and the RNA-seq data (Fig. 4).

### The expression of HMGCS1, HMGCR and the concentration of free cholesterol were upregulated in high glucose-induced HUVECs

**Fig. 5:**
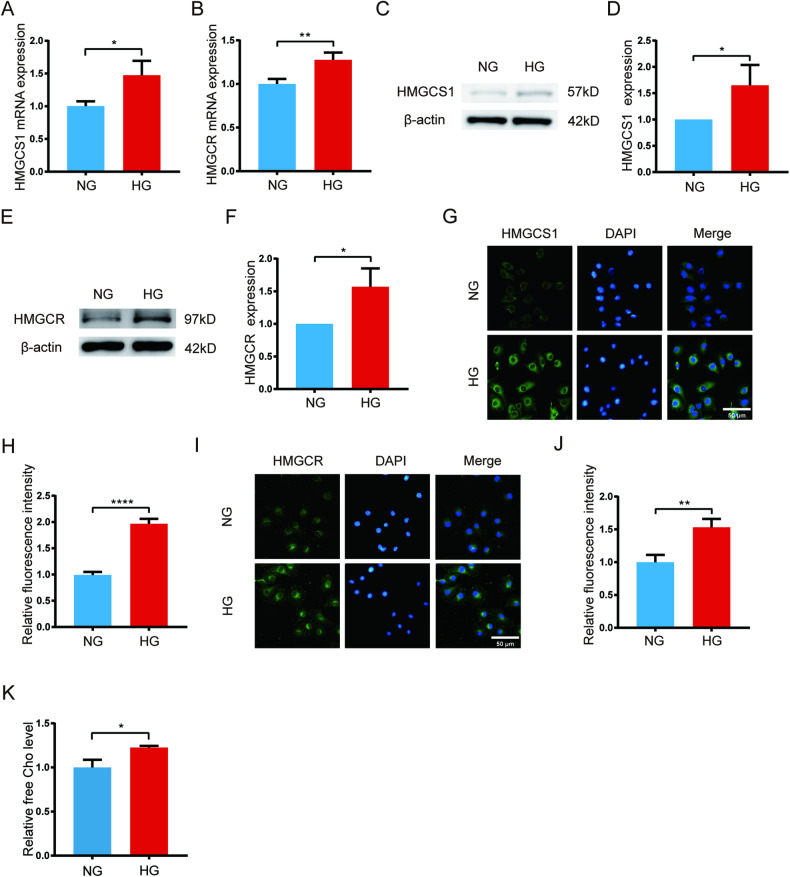
The expression of HMGCS1, HMGCR and the concentration of free cholesterol were upregulated in high glucose-induced HUVECs. **A**, **B** qPCR analysis of the mRNA level of HMGCS1 and HMGCR after high glucose treatment for 48 h. **C**–**F** Western blot and quantification of the protein level of HMGCS1 and HMGCR after high glucose treatment for 72 h. **G**–**J** Immunofluorescence of HMGCS1 and HMGCR and quantification of fluorescence intensity after high glucose treatment for 72 h. **K** Free cholesterol assay 72 h after high glucose treatment. Each experiment was replicated at least thrice, and representative images were shown. Quantification was done using a two-tailed unpaired Student’s *t* test. The results are presented as the mean ± SEM, *p < 0.05, **p < 0.01, ****p < 0.0001 vs controls.

Cholesterol exists partially as FC within cells and excessive FC accumulation has been demonstrated to induce cell apoptosis [16]. To determine whether cholesterol is over-synthesized in HUVECs under high glucose conditions, we detected the expression of HMGCS1 and HMGCR using qPCR, western blot and immunofluorescence. Additionally, the FC levels were measured using a commercial kit. After 48 h of treatment, the mRNA levels of HMGCS1 and HMGCR were found to be elevated by 47% and 28%, respectively, in the high glucose (HG) group (Fig. 5A, B). Furthermore, the protein levels of HMGCS1 and HMGCR increased by 65% and 57%, respectively, after 72 h of high glucose treatment (Fig. 5C–F). Immunofluorescence analysis also demonstrated higher fluorescence intensity in the HG group after 72 h of treatment (Fig. 5G–J). Moreover, the FC level in the high glucose-induced group was 23% higher than that in the control group after 72 h of treatment (Fig. 5K). These results collectively indicated that high glucose exacerbates the accumulation of free cholesterol in HUVECs by upregulating the expression of HMGCS1 and HMGCR, thereby promoting cholesterol biosynthesis.

### PKCδ knockdown decreased the expression of HMGCS1, HMGCR and the concentration of free cholesterol in high glucose-treated HUVECs

**Fig. 6:**
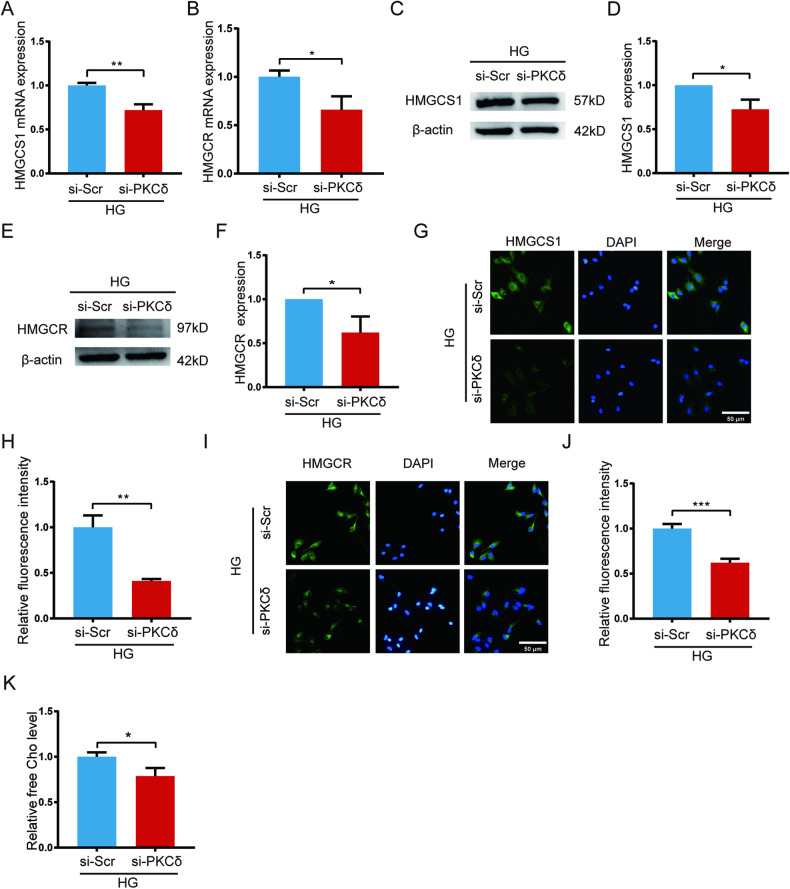
PKCδ knockdown by siRNA decreased HMGCS1, HMGCR expression and free cholesterol level in high glucose-treated HUVECs. **A**, **B** qPCR analysis of the mRNA level of HMGCS1 and HMGCR after transfection for 72 h. **C**–**F** Western blot and quantification of the protein level of HMGCS1 and HMGCR 72 h after transfection. **G**–**J** Immunofluorescence of HMGCS1 and HMGCR and quantification of fluorescence intensity after transfection for 72 h. **K** Free cholesterol assay 72 h after transfection. Each experiment was replicated at least thrice and representative images were shown. Quantification was done using a two-tailed unpaired Student’s *t* test. The results are presented as the mean ± SEM, *p < 0.05, **p < 0.01, ***p < 0.001 vs controls.

To verify the findings of the RNA-seq analysis, we examined the expression of HMGCS1 and HMGCR at the transcriptional level after PKCδ knockdown. Consistently, both HMGCS1 and HMGCR were downregulated by 28% and 34%, respectively, in the si-PKCδ group 72 h after transfection (Fig. 6A, B). At the protein level, western blot analysis performed 72 h after transfection revealed that si-PKCδ transfection resulted in a 27% and 37% decrease in the expression of HMGCS1 and HMGCR, respectively (Fig. 6C–F). Immunofluorescence targeting HMGCS1 and HMGCR further confirmed their reduced expression 72 h after transfection (Fig. 6G–J). To assess the effect of PKCδ knockdown on the FC concentration in HUVECs, the FC level was measured 72 h after transfection using a commercial kit. Notably, the si-PKCδ group exhibited a 23% decrease in the FC level compared to the si-Scr group (Fig. 6K). These findings illustrated that PKCδ knockdown downregulates the expression of two crucial enzymes involved in cholesterol production and reduces the FC level, suggesting that PKCδ knockdown may lower the intracellular FC level by inhibiting cholesterol synthesis.

### PKCδ inhibition decreased the expression of HMGCS1, HMGCR and the concentration of free cholesterol in high glucose-treated HUVECs

We further investigated the effect of rottlerin, a PKCδ activity inhibitor, on the expression of HMGCS1 and HMGCR, as well as the FC level. qPCR analysis revealed that, compared to HUVECs treated with the vehicle (DMSO), rottlerin treatment under high glucose conditions led to a 36% and 31% decrease in the mRNA expression of HMGCS1 and HMGCR, respectively (Supplementary Figs. S2A, B). western blot analysis showed a reduction of 35% in HMGCS1 and 41% in HMGCR protein levels after 24 h of rottlerin treatment (Supplementary Figs. S2C–F). Immunofluorescence analysis of HUVECs treated with rottlerin exhibited a 35% and 45% decrease in the fluorescence intensity of HMGCS1 and HMGCR, respectively (Supplementary Figs. S2G–J). Furthermore, the FC level was 18% lower in rottlerin-treated HUVECs compared to the vehicle-treated group after 24 h of treatment (Supplementary Figs. S2K). Interestingly, PKCδ inhibition under normal glucose also decreased HMGCS1 and HMGCR expression and FC levels (Supplementary Figs. S5A–D).

### Cholesterol restores high glucose-induced apoptosis in si-PKCδ-treated HUVECs and rottlerin-treated HUVECs

**Fig. 7:**
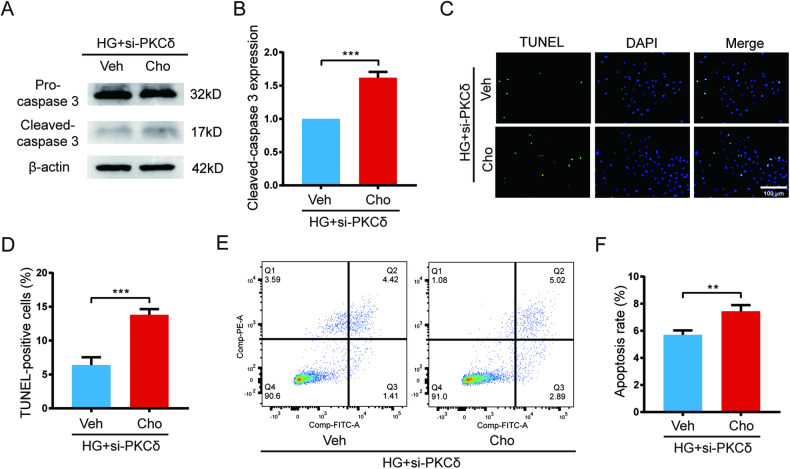
Cholesterol restored high glucose-induced apoptosis in si-PKCδ-treated HUVECs. **A**, **B** Western blot and quantification of cleaved-caspase 3 after cholesterol treatment for 24 h in si-PKCδ-transfected HUVECs. **C**, **D** TUNEL assay and quantification of TUNEL-positive cells after cholesterol treatment for 24 h in si-PKCδ-transfected HUVECs. **E**, **F** Flow cytometry assay and quantification of apoptotic cells after cholesterol treatment for 24 h in si-PKCδ-transfected HUVECs. Each experiment was replicated at least thrice and representative images were shown. Quantification was done using a two-tailed unpaired Student’s *t* test. The results are presented as the mean ± SEM, **p < 0.01, ***p < 0.001, vs controls.

Based on the aforementioned results, it can be suggested that high glucose upregulates PKCδ, which in turn upregulates HMGCS1 and HMGCR, stimulates cholesterol biosynthesis, and attenuates cell apoptosis. To confirm the effect of cholesterol on HUVEC apoptosis, a recovery experiment was conducted. After si-PKCδ transfection for 48 h, cholesterol (10 mg/dL) or an equivalent amount of vehicle (ethanol) was added to the medium and cultured for an additional 24 h before detection. Western blot analysis revealed a 62% increase in the expression of cleaved caspase 3 in the cholesterol-treated group compared to the control group (Fig. 7A, B). The percentage of TUNEL-positive cells also increased from 7% to 14% following cholesterol treatment (Fig. 7C, D). Additionally, flow cytometry analysis showed that cholesterol-treated HUVECs had a higher proportion of apoptotic cells (Fig. 7E, F).

Similarly, the effect of cholesterol on apoptosis in rottlerin-treated HUVECs under high glucose conditions was examined. After 24 h of rottlerin treatment, cholesterol or the solvent was added to the medium, and the cells were cultured for an additional 24 h before detection. The cholesterol-treated group exhibited a 39% higher expression of cleaved caspase 3 compared to the vehicle-treated group (Supplementary Figs. S3A, B). The percentage of TUNEL-positive cells also increased from 6% to 12% following cholesterol treatment (Supplementary Figs. S3C, D). Moreover, cholesterol treatment doubled the ratio of apoptotic cells, including early and late apoptotic cells, from 5% to 10% (Supplementary Figs. S3E, F).

### HMGCR inhibition accelerated wound healing of STZ-induced diabetic mice

**Fig. 8:**
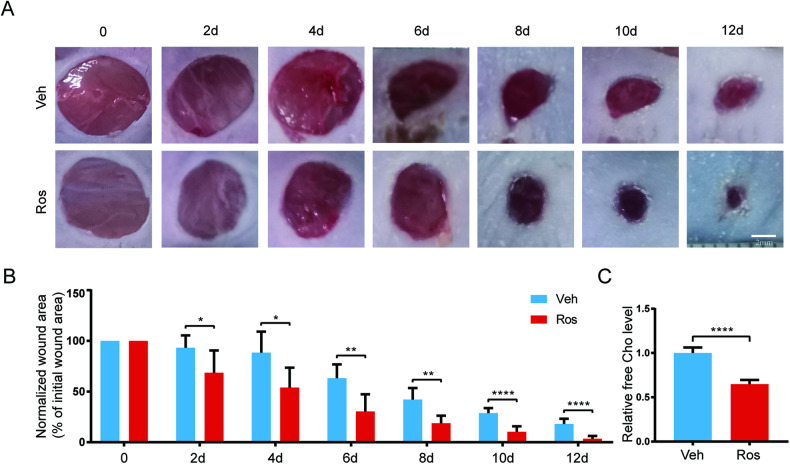
HMGCR inhibition accelerated wound healing of STZ-induced diabetic mice. **A**, **B** Images and quantification of wound area in rosuvastatin calcium-treated group and control group. **C** The free cholesterol level of skin tissue after rosuvastatin calcium treatment for 12 days. Each experiment was replicated at least thrice and representative images were shown. Quantification was done using a two-tailed unpaired Student’s *t* test. The results are presented as the mean ± SEM, *p < 0.05, **p < 0.01, ****p < 0.0001 vs controls.

The previous results have demonstrated that the PKCδ-HMGCS1/HMGCR pathway activation and increased free cholesterol levels mediate high glucose-induced HUVEC apoptosis. To further elucidate the effect of PKCδ-HMGCS1/HMGCR pathway inhibition on endothelial apoptosis and wound healing in vivo, an STZ-induced diabetic mice model was employed. Circular skin wounds were created on the backs of the mice following the formation of the diabetic model. The experimental group received rosuvastatin calcium (5 mg/kg i.p.), while the control group received an equivalent amount of vehicle. Images of the wounds were captured every two days, and a significantly higher healing rate was observed in the rosuvastatin calcium-treated group (Fig. 8A, B). Skin tissues surrounding the wounds were harvested at 12 days, and the free cholesterol level was measured. Rosuvastatin calcium treatment was found to downregulate the free cholesterol level in the skin of diabetic mice (Fig. 8C).

## Discussion

DFU is associated with the activation of many pathways, including the polyol pathway, nonenzymatic glycation pathway, advanced glycation end products pathway, reactive oxygen pathway, and the diacylglycerol (DAG)-protein kinase C (PKC) pathway [6]. The DAG-PKC pathway is activated under high glucose due to increased dihydroxyacetone phosphate, which is the glycolytic intermediate and promotes DAG synthesis. It has been demonstrated that high glucose levels activate PKC, impairing the normal function of human dermal microvascular endothelial cells, human dermal fibroblasts, and keratinocytes [34–36]. Interestingly, PKC activity was even higher under intermittent high glucose, which mimics the physical context more closely [37]. PKC isoforms can be divided into three subgroups, conventional PKCs (PKCα, PKCβI, PKCβII, and PKCγ), novel PKCs (PKCδ, PKCε, PKCη, and PKCθ), and atypical PKCs (PKCζ and PKCλ/ι). Only conventional PKCs and novel PKCs can be activated by DAG [6]. While current studies have primarily focused more on the activation of PKCβ in mediating DFUs, our study demonstrated the activation of PKCδ isoform in high glucose-treated HUVECs and skin tissues from diabetic mice, indicating a possible role of PKCδ in mediating diabetic foot ulcers [38].

Diabetic foot ulcers are associated with the dysfunction of several types of cells including endothelial cells, fibroblasts, and keratinocytes. Our previous studies have illustrated potential mechanisms underlying impaired collage deposition and disordered angiogenesis in diabetic wound healing [39–41]. Programmed cell death, which includes apoptosis, autophagy, pyroptosis, necroptosis, ferroptosis, and cuproptosis, has been shown to play an important role in diabetic wound healing [42, 43]. Diabetes causes endothelial cell apoptosis, which impairs angiogenesis and wound healing [5, 44]. PKCδ has been reported to mediate high glucose-induced apoptosis in several cell types [23, 45]. In our study, we inhibited PKCδ in high glucose-cultured endothelial cells using siRNA and rottlerin. We found PKCδ inhibition ameliorated endothelial apoptosis, indicating PKCδ as a mediator of high glucose-induced endothelial apoptosis. Although rottlerin has been used as a relatively specific inhibitor of PKCδ, it may also inhibit or activate other signal pathways [46]. It has been shown to play a dual role in regulating cell proliferation and apoptosis, possibly dependent on cell type, treatment period, and dosage. For example, 10 μM rottlerin alone promotes apoptosis, but 5 μM rottlerin ameliorates etoposide-induced apoptosis in C6 glioma cells. Furthermore, rottlerin was reported to inhibit angiogenesis in a dose-dependent manner [47]. The application of rottlerin as potential pharmaceutical for diabetic wounds needs further exploration.

DM, especially type 2 DM, is usually associated with abnormal lipid metabolism, manifested as mixed dyslipidemia [48]. In recent years, the role of altered lipid metabolism in diabetic complications has received increasing attention [49]. The lipid-lowering therapy has been shown to be effective in the management of microvascular complications of diabetes [50]. Statin treatment, in particular, has been reported to accelerate diabetic wound healing [51]. In our study, we identified 424 DEGs with a standard of fold change >1.2 and *q* < 0.05. KEGG pathway enrichment was performed, with an emphasis on lipid metabolism pathways. Although only two genes (HMGCS1 and HMGCR) were downregulated by si-PKCδ, these genes are key enzymes involved in cholesterol synthesis, suggesting PKCδ as an upstream promoter. To validate the results of RNA-seq, we employed si-PKCδ and rottlerin to downregulate PKCδ and examined the mRNA and protein levels of HMGCS1 and HMGCR. Both methods demonstrated reduced expression of HMGCS1 and HMGCR. Notably, both approaches also decreased the level of free cholesterol, indicating that PKCδ downregulation reduces free cholesterol levels in HUVECs by limiting cholesterol biosynthesis.

As an important component of cell membranes, cholesterol plays an important role in regulating cellular and systemic functions [52]. Under normal physiological conditions, cholesterol metabolism maintains homeostasis by balancing biosynthesis, absorption, export and esterification. However, the balance is perturbed in DM, resulting in hyperlipidemia [53]. In type 2 diabetic patients, cholesterol synthesis increases as blood glucose levels rise, while insulin therapy lowers fasting plasma cholesterol [54, 55]. Nevertheless, patients with type 1 DM show lower cholesterol synthesis but higher absorption [56]. Abnormalities in cholesterol metabolism contribute to delayed wound healing. High-density lipoprotein (HDL) reduced inflammation, promoted angiogenesis, and reduced amputation risk, while low-density lipoprotein inhibited endothelial proliferation and angiogenesis [57–59]. Cholesterol overload, especially free cholesterol, also leads to cell apoptosis, which is a potential mechanism underlying impaired diabetic wound healing [16, 17, 60, 61]. It has been reported that high glucose promotes cholesterol accumulation in several cell types by increasing biosynthesis or impairing the balance between influx and efflux [62–64]. Our study found elevated levels of free cholesterol in both high glucose-treated HUVECs and skin tissues from diabetic mice, suggesting a basis for cholesterol-lowing therapy in diabetic foot ulcers. Moreover, after PKCδ knockdown or inhibition under high glucose, we added exogenous cholesterol to the medium. Cholesterol restored high glucose-induced apoptosis and eliminated the protective effect of PKCδ downregulation. These findings indicated that PKCδ mediates high glucose-induced HUVECs apoptosis by promoting cholesterol biosynthesis.

From our study, we may conclude that the inhibition of the PKCδ-HMGCS1/HMGCR pathway alleviates high glucose-induced endothelial apoptosis and represent a potential therapeutic strategy for diabetic foot ulcers [46]. Evidence showed that rottlerin inhibited the proliferation of keratinocytes, collagen production in dermal fibroblasts, and angiogenesis of endothelial cells [47, 65, 66]. While these effects are beneficial to the treatment of proliferative diseases such as psoriasis and tumors, they may hinder wound healing [67]. With advancements in delivery materials, siRNA therapeutics become more tissue-specific and efficient [68]. Local delivery strategies, including microparticles, scaffolds, electrospun fibers, hydrogels, and surface coatings, could be considered for the administration of si-PKCδ on diabetic wound [68]. Additionally, δV1-1, a relatively specific inhibitor of PKCδ that suppresses its translocation, could be employed [69]. It comprises 10 amino acids (8-17, SFNSYELGSL) and is typically conjugated to a TAT carrier peptide. δV1-1 has been utilized in both in vitro and in vivo studies and may aid wound healing when applied topically. However, since PKCδ acts as an upstream kinase regulating numerous essential reactions, direct inhibition of PKCδ may have unanticipated effects on normal cell signaling and metabolism. Therefore, we directly suppressed a downstream and relatively specific gene, HMGCR, and downregulated the executive metabolite, cholesterol. Statins, a class of drugs that inhibits the activity of HMGCR, have been widely used in clinical practice to lower blood lipids. Several stains, including simvastatin, atorvastatin, pravastatin, and others, have been proved to benefit wound healing [51]. In our study, we administrated intraperitoneal injections of a more potent stain, rosuvastatin calcium, to STZ-induced type 2 diabetic mice. Compared with the control group (saline), rosuvastatin calcium treatment significantly accelerated the wound healing process. Thus, we may propose that statins could be a good choice for patients with both hypercholesteremia and diabetic foot ulcers. However, statins may also lead to the progression of diabetes [70]. More clinical evidence is needed to evaluate the risks and benefits of statins in patients with diabetic foot ulcer.

Unanswered questions in the present study include the mechanism of PKCδ activation under diabetes, the sites of PKCδ phosphorylation, and the regulatory mechanism between PKCδ and HMGCS1/HMGCR, all of which require additional investigation.

## Conclusion

In our study, we demonstrated a pro-apoptotic role of PKCδ in mediating high-glucose-induced HUVECs apoptosis. We further investigated the downstream targets of PKCδ and identified HMGCS1 and HMGCR as key genes that are upregulated by PKCδ. The upregulation of HMGCS1 and HMGCR leads to increased cholesterol biosynthesis in HUVECs, exacerbating their apoptosis and contributing to the delayed healing of diabetic foot ulcers. These findings have important implications for the development of innovative strategies aimed at accelerating the healing process of diabetic foot ulcers. One such strategy could involve the topical or systemic administration of HMGCR inhibitors, which have the potential to modulate cholesterol biosynthesis and improve wound healing outcomes in diabetic individuals.

## Method

### Chemicals and antibodies

The Chemicals used in this study included: Rottlerin (#S7862, Selleck), Cholesterol (#HY-N0322, MedChemExpress), DMSO (#HY-Y0320, MedChemExpress), Streptozocin (#HY-13753, MedChemExpress), Rosuvastatin Calcium (#HY-17504, MedChemExpress).

The antibodies used in this study included: PKCδ polyclonal antibody (#19132-1-AP, Proteintech), Caspase 3/p17/p19 Polyclonal antibody (#19677-1-AP, Proteintech), HMGCS1 Rabbit pAb (#A3916, ABclonal Techonlogy), HMGCR Rabbit pAb (#A1633, ABclonal Techonlogy), GAPDH Monoclonal antibody (#60004-1-Ig, Proteintech), Beta Actin Monoclonal antibody (#66009-1-Ig, Proteintech), HRP-conjugated Affinipure Goat Anti-Rabbit IgG(H + L) (#SA00001-2, proteintech), HRP-conjugated Affinipure Goat Anti-Mouse IgG(H + L) (#SA00001-1, Proteintech), CoraLite488-conjugated Goat Anti-Rabbit IgG(H + L) (#SA00013-2, Proteintech).

### Cell culture and treatment

Human umbilical vein endothelial cells (HUVECs) were acquired from the Cell Bank of the Chinese Academy of Science (Shanghai, China). The cells were cultured in normal glucose (NG, 5.5 mM)-DMEM (#PM150220, Procell Life Science&Technology), supplemented with 10% FBS (#164210, Procell Life Science&Technology) and 1% antibiotics, at 37 °C in a 95% air–5% CO2 atmosphere. High glucose (HG, 22.5 mM)-DMEM (#PM150210, Procell Life Science&Technology), supplemented with 10% FBS and 1% antibiotics, was employed to mimic a diabetic condition.

### Animal and experimental design

45 specific pathogen-free C57BL/6 mice (male, 6 weeks old) and 8 db/db mice (male, seven weeks old) were acquired from SHULAIBAO Biotech (Hubei, China) and bred in conventional animal facilities in a temperature- and humidity-controlled environment with a 12-h light/dark cycle. Animal care and experimental procedures were approved by the Principles of Animal Use Committee (NIH Publications No. 8023, revised 1978). PKCδ mRNA and protein expression were measured in two type 2 diabetic mice models: streptozotocin (STZ)-induced diabetes and db/db mouse model. 22 C57BL/6 mice were fed a high-fat diet for four weeks. 14 C57BL/6 mice were intraperitoneally injected with STZ to create type 2 diabetes mice model and 8 C57BL/6 mice were injected with vehicle (citrate buffer) as control. Two weeks after the STZ-induced diabetes mouse model creation, euthanasia was performed followed by back skin tissue harvesting. All db/db mice were fed a normal diet, along with 8 C57BL/6 mice as control. After two weeks, back skin tissues were harvested. The other 15 C57BL/6 mice were used to create STZ-induced diabetes mice model to test the effect of HMGCR inhibitor. Circular wounds were made on the back of STZ-induced diabetes mice. Rosuvastatin calcium (5 mg/kg i.p.) or equal amount of vehicle was given daily for two weeks. Photos were taken every 2 days and wound-surrounding skin was harvested at 12d. All C57BL6/ mice were randomly allocated and blinding was done.

### STZ-induced diabetes mouse model creation

To create the STZ-induced diabetes model, C57BL/6 mice were fed a high-fat diet for 4 weeks, followed by a one-time intraperitoneal streptozotocin injection (100 mg/kg in 0.1 mol/L citrate buffer, pH 4.5) after an overnight fast. After 3 days, non-fasted glucose was measured from tail blood, and successful induction of diabetes was confirmed if blood glucose was over 16.67 mM.

### siRNA transfection

siRNA against the human PKCδ gene (si-PKCδ) and the scramble siRNA (si-Scr) were designed and synthesized by GenePharma (Shanghai, China). The sequences are as follows: si-PKCδ, -GCU GCC AUC CAC AAG AAA UTT- (sense) and -AUU UCU UGU GGA UGG CAG CTT- (antisense); si-Scr, -UUC UCC GAA CGU GUC ACG UTT- (sense) and -ACG UGA CAC GUU CGG AGA ATT- (antisense); Transient transfection was performed using Lipo8000 (#C0533, Beyotime) according to the manufacturer’s instructions.

### qPCR analysis

qPCR was performed to access the mRNA expression levels of PKCδ, HMGCS1, and HMGCR in cultured HUVECs and mouse skin tissue. Total RNA was isolated using TRIZOL (#R401-01, Vazyme Biotech) and reverse-transcribed into cDNA using HiScript III RT SuperMix for qPCR (#R323, Vazyme Biotech) following the manufacturer’s protocol. qPCR was implemented with ChamQ SYBR qPCR Master Mix (#Q311-02, Vazyme Biotech,). The primers used were as follows: Human PKCδ, -GTG CAG AAG AAG CCG ACC AT- (forward) and -CCC GCA TTA GCA CAA TCT GGA- (reverse); Human HMGCS1, -GCT CTT GGG ATG GAC GGT ATG C- (forward) and -ACT GCT CCA ACT CCA CCT GTA GG- (reverse); Human HMGCR, -AGC ATT GGC AGC AGG ACA TCT TG- (forward) and -CGG GCT ATT CAG GCT GTC TTC TTG- (reverse); Mouse PKCδ, -GGC TCC CTG CAA GTT GAG G- (forward) and -ACA CGG CCT TCA TAG ATG TGG- (reverse); Mouse HMGCS1, -CGG ATC GTG AAG ACA TCA ACT C- (forward) and -CGC CCA ATG CAA TCA TAG GAA- (reverse); Mouse HMGCR, -TGT TCA CCG GCA ACA ACA AGA- (forward) and -CCG CGT TAT CGT CAG GAT GA- (reverse); All primers were obtained from Sangon Biotech (Shanghai, China). The expression of glyceraldehyde-3-phosphate dehydrogenase (GAPDH) was used as a normalization control.

### RNA sequencing

RNA samples from cultured HUVECs were extracted and assessed for quality by BGI company (Shenzhen, China). RNA-sequencing (RNA-Seq) was executed on a commercially available service (#F19FTSCCWLJ6421, BGI, Wuhan, China). Briefly, a certain amount of RNA was denatured and mRNA was enriched with oligo (dT) magnetic beads. Subsequently, mRNA fragmentation, cDNA synthesis, end-repair, 3′-adenylation, and fork-tailed adapter ligation were performed. After PCR amplification, library quality control and circulation were carried out. Finally, Single-stranded circle DNA molecules were replicated, generating a DNA nanoball (DNB). DNBs of sufficient quality were then loaded into patterned nanoarrays using high-intensity DNA nanochip technology and sequenced using combinatorial Probe-Anchor Synthesis. The FASTQ data from the sequencing has been deposited in the CNGB Nucleotide Sequence Archive (CNSA) repository (CNP0004333).

### Western blot analysis

Cells were lysed using RIPA lysis buffer (# P0013K, Beyotime) supplemented with protease inhibitors and skin tissue was ground with a tissue lyser (#KZ-II, Servicebio). The protein content was measured with a BCA kit (#P0010, Beyotime). The lysate (10-20 ug protein) was separated by SDS- PAGE, transferred to a polyvinylidene fluoride (PVDF) membrane, and blocked with 5% skim milk. The membranes were probed with specific primary antibodies overnight at 4 °C. Subsequently, the membranes were incubated in HRP-conjugated secondary antibody for 2 h at room temperature and visualized with an ECL reagent (# BL520A, Biosharp). Protein content quantification was evaluated with Image J software (NIH, USA).

### Immunofluorescence

HUVECs were plated in 12-well plates with a coverslip over each well and incubated in growth medium. After various treatments, cells were washed twice with cold phosphate-buffered saline (PBS), fixed with 4% formaldehyde for 15 min, and permeabilized with 0.1% Triton X-100 for 10 min at room temperature. The cells were then washed thrice with PBS and blocked with 1% bovine serum albumin in PBS for 1 h at room temperature. Primary antibodies diluted in a 1:100 blocking solution were added to each coverslip and incubated overnight at 4 °C. After washing thrice with PBS, the coverslips were incubated with CoraLite488-conjugated goat anti-rabbit antibody diluting at 1:200 for 1 h at 37 °C in the dark. Then the coverslips were rinsed thrice with PBS, followed by DAPI staining for 5 min. The fluorescence intensity of the cells mounted in 90% glycerol PBS was examined using a fluorescence microscope. Image J software (NIH, USA) was used to evaluate fluorescence intensity.

### TUNEL assay

TUNEL assay was performed using a commercial kit (# E-CK-A320, Elabscience). Briefly, cells were washed with cold PBS thrice, fixed with 4% formaldehyde for 15 min, permeabilized with 0.1% Triton X-100 for 10 min at room temperature and stained for 1 h at 37 °C, followed by DAPI staining. Cells were visualized under a fluorescence microscope and assessed with Image J software (NIH, USA). The TUNEL-positive ratio was calculated as the ratio of positively stained cells to the total number of cells.

### Flow cytometry apoptosis assay

Cells were stained with Annexin-V-FITC and PI using a commercial kit (#C1062L, Beyotime) to access apoptosis. (AnnexinV-FITC)^-^/PI^-^ cells are normal cells, (AnnexinV-FITC)^−^/PI^+^ cells are necrotic cells, (AnnexinV-FITC)^+^/PI^−^ and (AnnexinV-FITC)^+^/PI^+^ cells are considered apoptotic cells. Results were analyzed using Flowjo (version 10).

### Free cholesterol assay

Free cholesterol (FC) was detected using a commercial kit (#BC1895, Solarbio) according to the manufacturer’s instructions. The absorbance at 500 nm was detected and the total protein level was used for normalization.

### Statistical analysis

Data are presented as the mean ± SEM from at least three independent experiments. Unpaired Student’s t-test was used to compare normally distributed quantitative variables. *p* < 0.05 was considered statistically significant. All figures were designed using GraphPad Prism 7.0 and Adobe Illustrator.

### Supplementary information

Supplementary Figure 1

Supplementary Figure 2

Supplementary Figure 3

Supplementary Figure 4

Supplementary Figure 5

Supplementary figure legends

## Data Availability

The raw data of RNA-sequencing has been deposited in the CNGB Nucleotide Sequence Archive (CNSA) repository (CNP0004333). The original data of the study are available from the corresponding author upon reasonable request.
