# Supplementary figures and images for: Upregulation of rate-limiting enzymes in cholesterol metabolism by PKCδ mediates endothelial apoptosis in diabetic wound healing
Source: Cell Death Discov. 2024 May 29;10:263. doi: 10.1038/s41420-024-02030-2 (PMC11137154; doi:10.1038/s41420-024-02030-2)

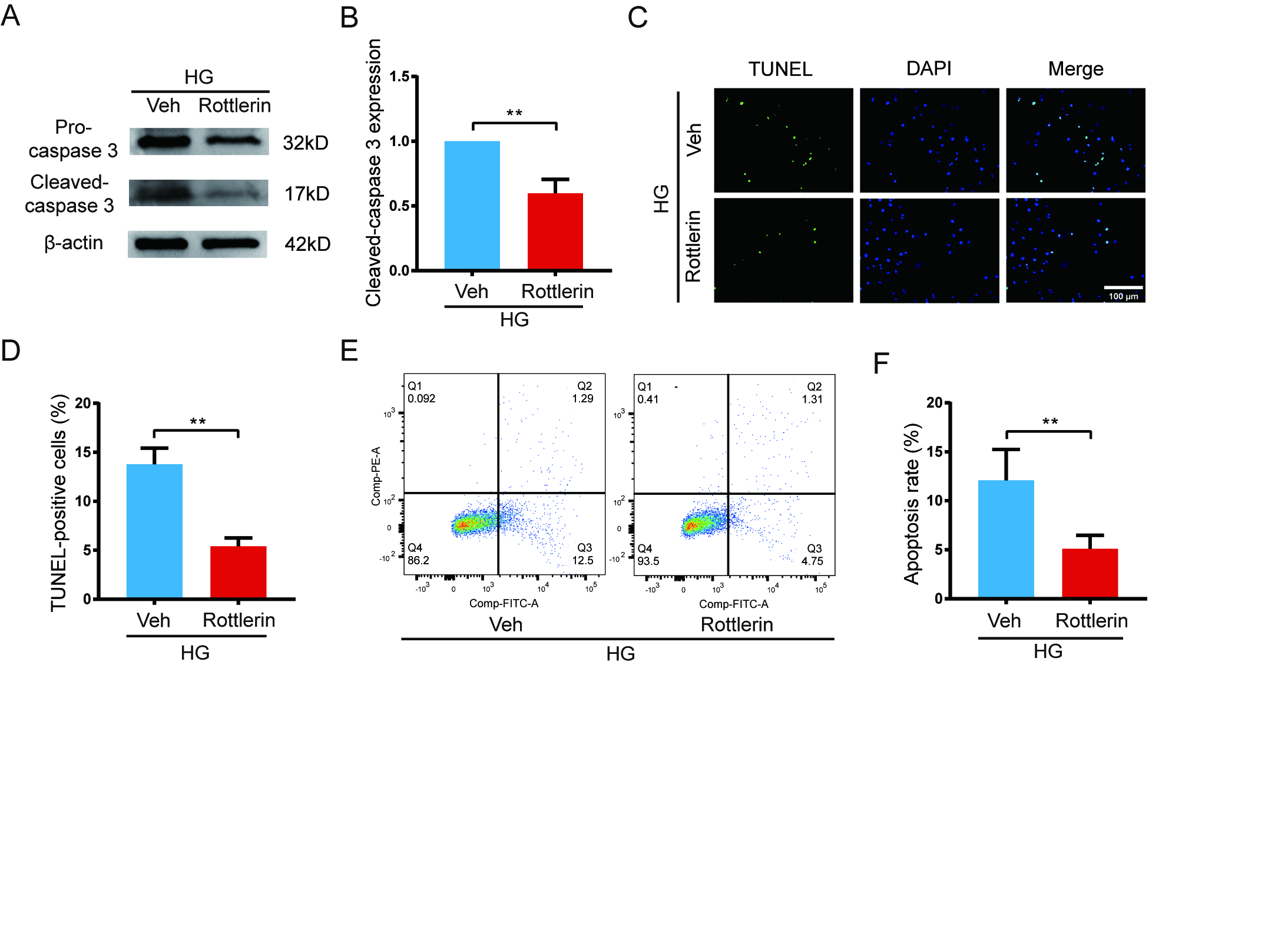

Supplement: Supplementary file 1 — Supplementary Figure 1 [file 41420_2024_2030_MOESM1_ESM.tif]

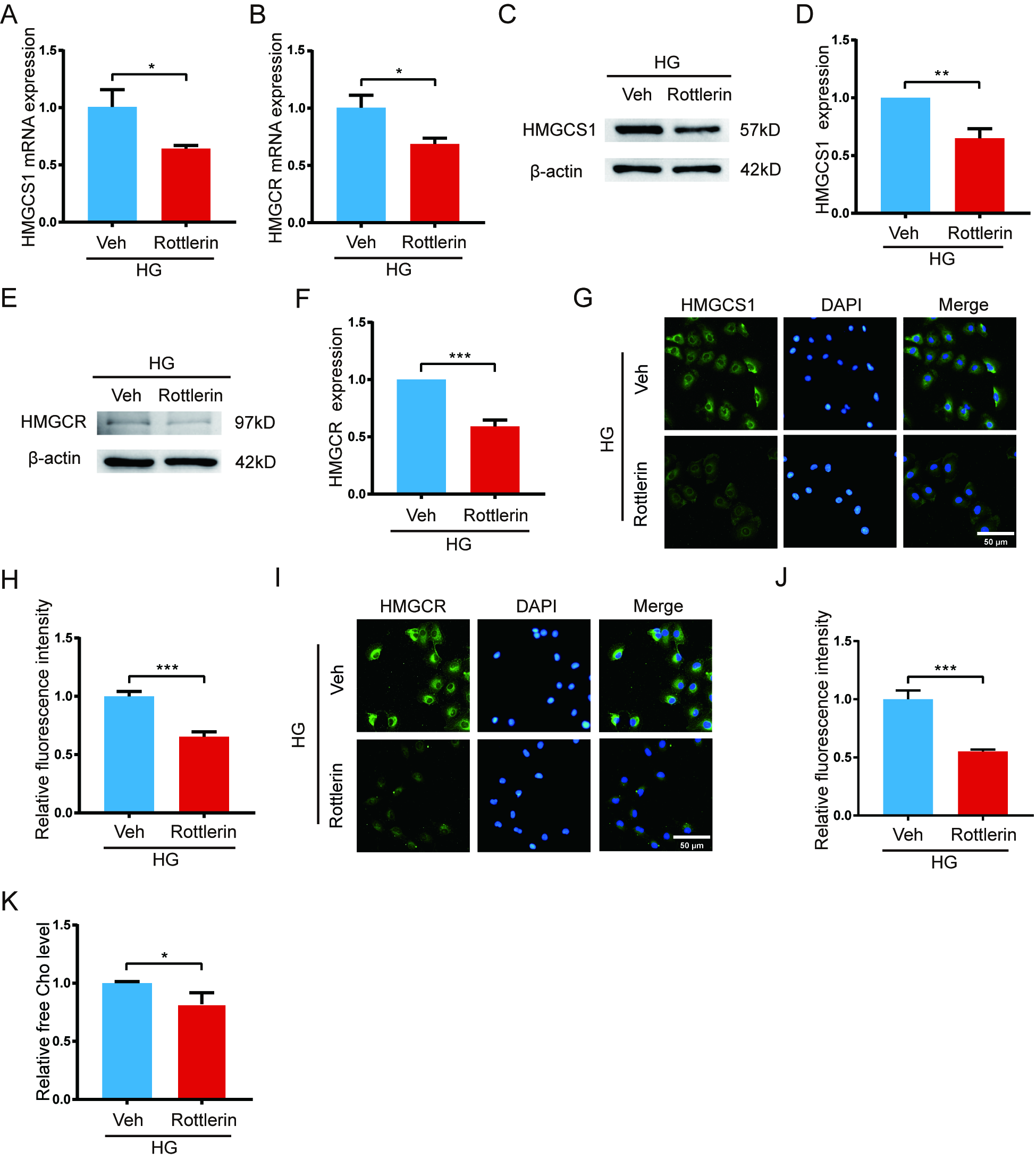

Supplement: Supplementary file 2 — Supplementary Figure 2 [file 41420_2024_2030_MOESM2_ESM.tif]

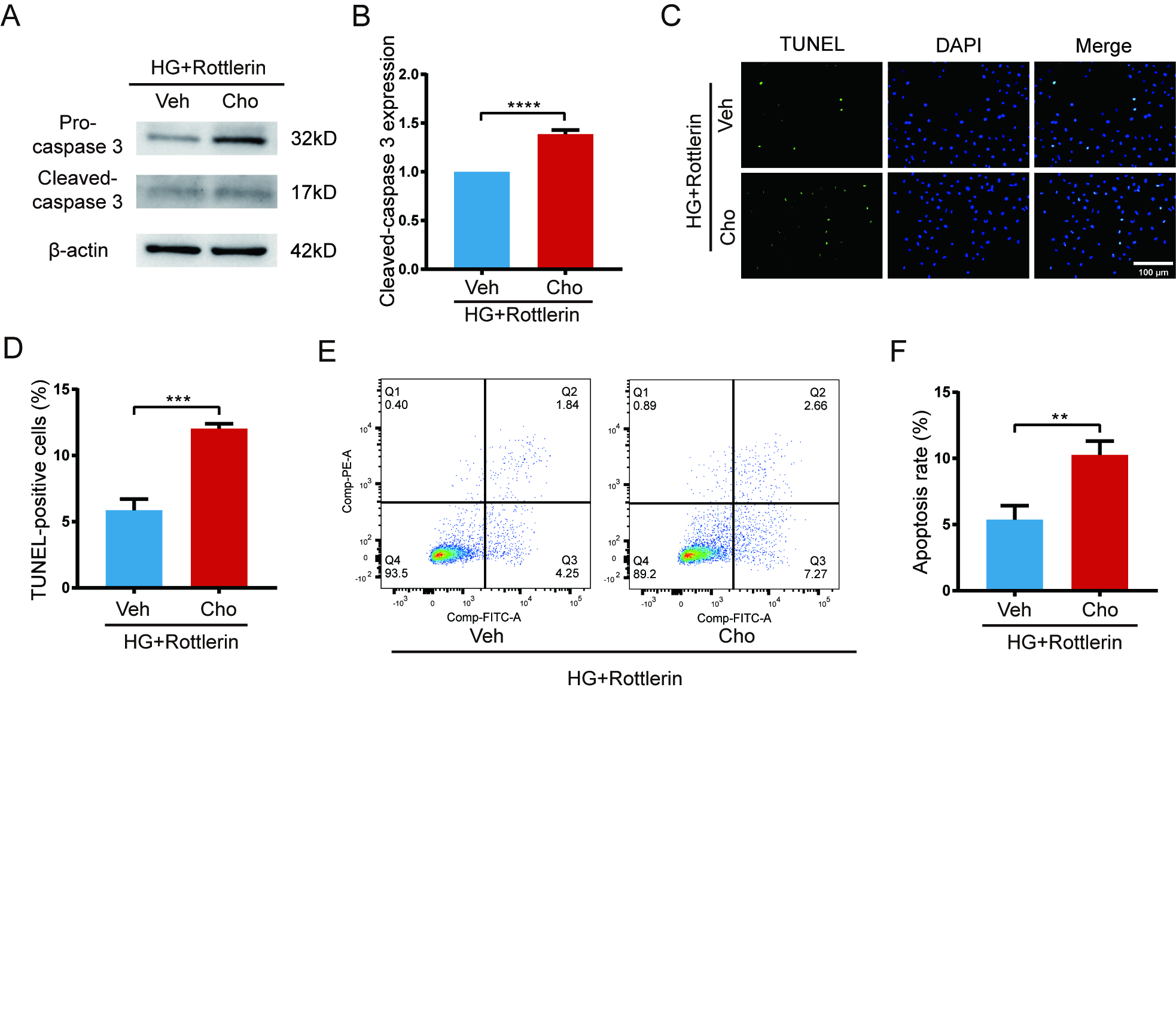

Supplement: Supplementary file 3 — Supplementary Figure 3 [file 41420_2024_2030_MOESM3_ESM.tif]

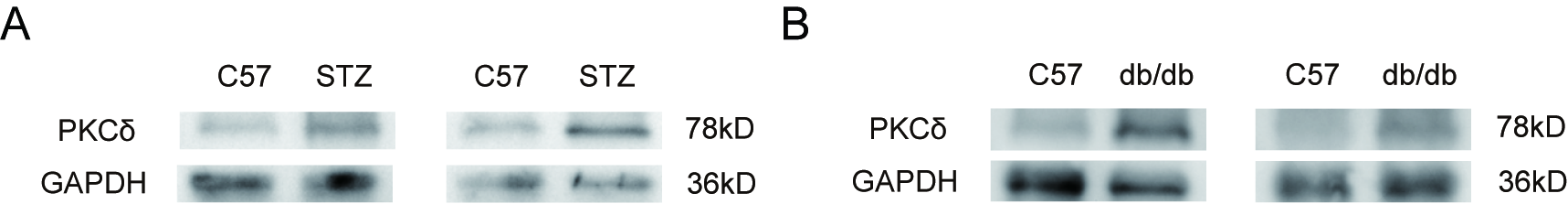

Supplement: Supplementary file 4 — Supplementary Figure 4 [file 41420_2024_2030_MOESM4_ESM.tif]

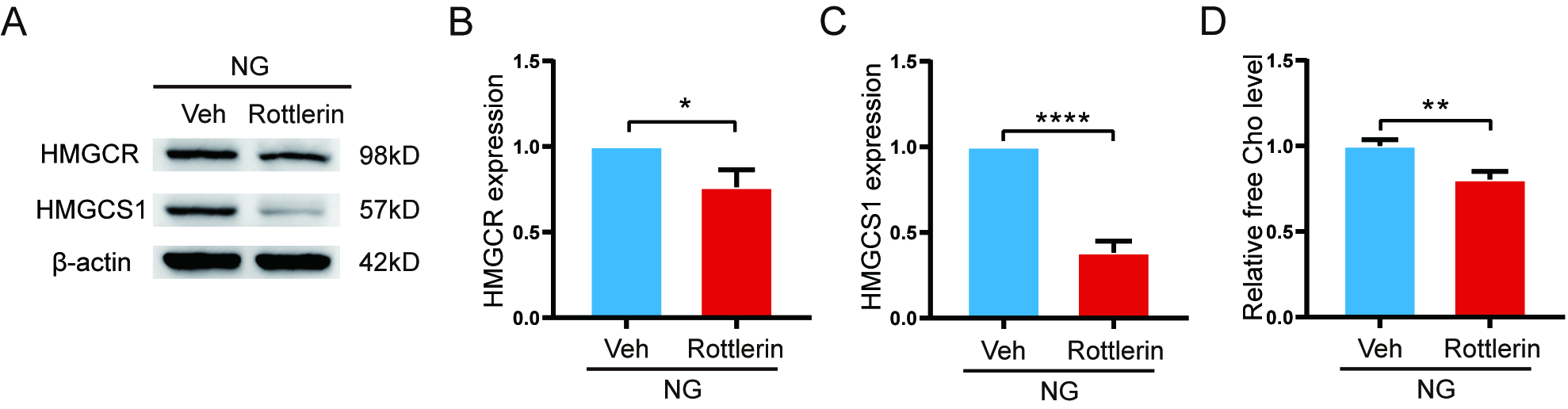

Supplement: Supplementary file 5 — Supplementary Figure 5 [file 41420_2024_2030_MOESM5_ESM.tif]
