# Supplementary material for: Upregulation of rate-limiting enzymes in cholesterol metabolism by PKCδ mediates endothelial apoptosis in diabetic wound healing
Source: Cell Death Discov. 2024 May 29;10:263. doi: 10.1038/s41420-024-02030-2 (PMC11137154; doi:10.1038/s41420-024-02030-2)
Supplement: Supplementary file 6 — Supplementary figure legends [file 41420_2024_2030_MOESM6_ESM.docx]

**Supplementary figure legends**

Figure S1. PKCδ inhibition by rottlerin ameliorated high glucose-induced HUVECs apoptosis. (A, B) Western blot analysis and quantification of cleaved-caspase 3 after rottlerin treatment for 48 h under high glucose. (C, D) TUNEL assay and quantification of the percentage of TUNEL-positive cells after rottlerin treatment for 48h. (E, F) Flow cytometry assay and quantification of the percentage of apoptotic (FITC-positive) cells after rottlerin treatment for 48 h. Each experiment was replicated for at least thrice and representative images were shown. Quantification was done using a two-tailed unpaired Student’s t-test. The results are presented as the mean ± SEM, ***p* < 0.01 vs controls.

Figure S2. PKCδ inhibition by rottlerin downregulated HMGCS1, HMGCR expression and free cholesterol level in high glucose-treated HUVECs. (A, B) qPCR analysis of HMGCS1 and HMGCR expression 24 h after rottlerin treatment. (C-F) Western blot analysis and quantification of HMGCS1 and HMGCR protein expression after rottlerin treatment for 24 h. (G-J) Immunofluorescence and quantification of HMGCS1 and HMGCR after rottlerin treatment for 24 h. (K) FC level detection 24 h after rottlerin treatment. Each experiment was replicated for at least thrice and representative images were shown. Quantification was done using a two-tailed unpaired Student’s t-test. The results are presented as the mean ± SEM, **p* < 0.05, ***p* < 0.01, ****p* < 0.001 vs controls.

Figure S3. Cholesterol restored high glucose-induced apoptosis in rottlerin-treated HUVECs. (A, B) Western blot and quantification of cleaved-caspase 3 after cholesterol treatment for 24 h in rottlerin-treated HUVECs. (C, D) TUNEL assay and quantification of TUNEL-positive cells after cholesterol treatment for 24 h in rottlerin-treated HUVECs. (E, F) Flow cytometry assay and quantification of apoptotic cells after cholesterol treatment for 24 h in rottlerin-treated HUVECs. Each experiment was replicated for at least thrice and representative images were shown. Quantification was done using a two-tailed unpaired Student’s t-test. The results are presented as the mean ± SEM, ***p* < 0.01, ****p* < 0.001, *****p* < 0.0001 vs controls.

Figure S4. The other two replicates of Figure 1G and 1J. (A) The other two replicates of Figure 1G. (B) The other two replicates of Figure 1J.

Figure S5. PKCδ inhibition by rottlerin downregulated HMGCS1, HMGCR expression and free cholesterol level in normal glucose-treated HUVECs. (A-C) Western blot analysis and quantification of HMGCS1 and HMGCR protein expression after rottlerin treatment for 24 h. (D) FC level detection 24 h after rottlerin treatment. Each experiment was replicated for at least thrice and representative images were shown. Quantification was done using a two-tailed unpaired Student’s t-test. The results are presented as the mean ± SEM, **p* < 0.05, ***p* < 0.01, *****p* < 0.0001 vs controls.
